# Rationally irresolvable disagreement

**DOI:** 10.1007/s11098-023-01933-7

**Published:** 2023-02-26

**Authors:** Guido Melchior

**Affiliations:** Department of Philosophy, University of Graz, Heinrichstrasse 26/5, 8010 Graz, Austria

**Keywords:** Deep disagreement, Disagreement, Rationality, Irresolvable disagreement

## Abstract

The discussion about deep disagreement has gained significant momentum in the last several years. This discussion often relies on the intuition that deep disagreement is, in some sense, rationally irresolvable. In this paper, I will provide a theory of rationally irresolvable disagreement. Such a theory is interesting in its own right, since it conflicts with the view that rational attitudes and procedures are paradigmatic tools for resolving disagreement. Moreover, I will suggest replacing discussions about deep disagreement with an analysis of rationally irresolvable disagreement, since this notion can be more clearly defined than deep disagreement and captures the basic intuitions underlying deep disagreement. I will first motivate this project by critically assessing the current debate about deep disagreement. I then detail the notions of rationality and resolvable disagreement which are crucial for a suitable theory of rationally irresolvable disagreement before sketching various instances of rationally irresolvable disagreement. Finally, I argue for replacing theories of deep disagreement with theories of rationally irresolvable disagreement, an approach that has significant advantages over existing theories of deep disagreement which focus on hinge propositions or fundamental epistemic principles.

## The discussion about deep disagreement

Debates over deep disagreement (DD) have always centered on its relationship to rationally irresolvable disagreement (RID). Fogelin (1985) summarizes his pioneering paper by characterizing DD as disagreements “which by their nature, are not subject to rational resolution.” The connection between DD and rational irresolvability is assumed by Adams (2005), Lynch (2010), and Aikin (2021), discussed by Ranalli (2021), and discussed but rejected by Lugg (1986), Feldman (2005), and Matheson (2021). Despite the central role of RID in DD, however, there is no explicit theory about RID yet on the market. Rather, philosophical discussions about DD currently focus on its metaphysical nature, with the two main schools defending either a hinge theory or a theory based on first epistemic principles. A theory of RID is interesting in its own right, but it can also be used to reframe the discussion about DD. In this paper, I will first, develop a theory of RID and, second, argue for replacing theories of DD by theories of RID (or at least by combining these theories).

Let me recall DD, as introduced by Fogelin (1985).^1^ Fogelin argues that DD arises when argumentative exchanges take place outside “a context of broadly shared beliefs and preferences.” Referring to Wittgenstein (1969), Fogelin (1985, 6) suggests that “for an argumentative exchange to be normal, there must exist shared procedures for resolving disagreements and that engaging in an “argumentative exchange, presupposes a background of shared commitments.” When the context of an argumentative exchange is neither normal nor nearly normal, then the preconditions for argument are not fulfilled because a shared background of beliefs and preferences is missing. Fogelin calls this *deep disagreement.* Fogelin refers to a specific Wittgensteinian concept of preconditions of argumentative exchange, when he notes “that deep disagreements cannot be resolved through the use of argument, for they undercut the conditions essential to arguing.” (1985, 7f). He continues to characterize DD as follows:

What is a deep disagreement? First let me say what I don’t mean by this notion. A disagreement can be intense without being deep. A disagreement can also be unresolvable without being deep. I can argue myself blue in the face trying to convince you of something without succeeding. The explanation might be that one of us is dense or pig-headed. And this is a matter that could be established beyond doubt to, say, an impartial spectator. But we get a very different sort of disagreement when it proceeds from a clash in underlying principles. Under these circumstances, the parties may be unbiased, free of prejudice, consistent, coherent, precise and rigorous, yet still disagree. And disagree profoundly, not just marginally. Now when I speak about underlying principles, I am thinking about what others (Putnam) have called framework propositions or what Wittgenstein was inclined to call rules. We get a deep disagreement when the argument is generated by a clash of framework propositions. (Fogelin, 1985, 8)

Fogelin’s description of DD fits into his Wittgensteinian picture on the preconditions of meaningful and successful argumentation. However, his overall picture on DD remains rather sketchy, and, consequently, a vivid discussion on DD emerged, which centers mainly on the following two questions:

- Q1: What is deep disagreement?
- Q2: Is deep disagreement resolvable or not?

The first question concerns the metaphysics of DD. Fogelin answers it by noting that “when we inquire into the source of a DD, we do not simply find isolated propositions […], but instead a whole system of mutually supporting propositions (and paradigms, models, styles of acting and thinking) that constitute, if I may use the phrase, a form of life.” (Fogelin, 1985, 9) On Fogelin’s view, DD not just concerns a particular proposition, it is a conflict between whole systems and ways of thinking. As for the second question, Fogelin is clearly pessimistic that it can be answered in the affirmative when he notes: “But if deep disagreements can arise, what rational procedures can be used for their resolution? The drift of this discussion leads to the answer NONE.” (Fogelin, 1985, 9).

Concerning both questions, in particular concerning Q1, different views have been defended: The two main alternatives are Wittgensteinian theories or hinge theories about the nature of DD and fundamental epistemic principle theories.^2^ Wittgenstein (1969) argues that there are some fundamental propositions, so called hinges, that we not only cannot justify but which we cannot rationally assess at all. Amongst those who have developed Wittgenstein’s thoughts on hinge commitments, there are a few distinctions that can be made. Some hinge theories are non-epistemic, arguing that hinge propositions are not open to epistemic assessment. These non-epistemic views can then be further divided into non-propositional views and non-belief views. Non-propositional views, as defended by Wright (1985) and Moyal-Sharrock (2004 and 2016) have it that hinges are not truth-apt and, therefore, they are not really propositions. The non-belief view is defended by Pritchard (2016 and forthcoming), who argues that hinge commitments are propositions, but they are not believable.^3^ Epistemic theories, on the other hand, take it that hinge commitments are open to epistemic assessment. An epistemic theory of hinge commitments is developed by Wright (2004 and 2014), who argues that hinge propositions are subject to rational evaluation because we are entitled to accept or trust hinge propositions.

Lynch (2010, 2016) and Kappel (2012, 2021) are proponents of the alternative view about DD. They argue that DD is disagreement about fundamental epistemic principles.^4^ For this type of views about DD, it is crucial to define fundamental epistemic principles. Lynch (2010) defines them as principles that can be justified only by means of epistemically circular arguments. Lagewaard (forthcoming) criticizes Lynch’s definition of DD since paradigmatic cases of DD only involve derived but not fundamental epistemic principles.^5^

There is more agreement about Q2 than about Q1. Most philosophers working on DD share Fogelin’s (1985) pessimistic view concerning its resolvability. Lynch (2010, 273), for example, refers to Wittgenstein’s metaphor when suggesting that where “there is deep epistemic disagreement over some fundamental principle, the disagreement has hit bedrock, the spade has turned.” Feldman (2005), one of the rare optimists concerning the resolvability of DD, argues that DD can be rationally resolved if the initially disagreeing parties suspend judgment about the target propositions.^6^

## From deep disagreement to rationally irresolvable disagreement

Let me start with a methodological point concerning DD. DD is not a commonsense notion of natural language, such as “knowledge” or “belief”. In cases of knowledge or belief, we can develop a theory and test it against our intuitions by considering controversial cases, as Gettier paradigmatically did with the JTB theory of knowledge. However, DD is also not a purely technical term that can be arbitrarily defined. Rather, DD can best be described as a semi-technical term. For analyzing such semi-technical terms, we often proceed as follows. A phenomenon is introduced, often by presenting paradigmatic cases, and through these examples we then form an intuitive grasp of the target phenomenon. After that, we can then analyze that notion within a systematic theoretical framework. However, different philosophers might have different intuitions about the target phenomenon. Moreover, they might think that different explanatory desiderata should be prioritized in theorizing about it. Accordingly, we might not always be able to judge which theory about the target phenomenon is true or false, but only whether those theories meet the specific explanatory desiderata.^7^

All of these points apply to the discussion about DD. Adherents of hinge theories might think that disagreement about hinge propositions *is* the target phenomenon, and, based on this intuition, develop their hinge accounts of DD. The same might hold *mutatis mutandis* for adherents of fundamental-epistemic-principles views. However, both parties might be right in that there is disagreement based on hinge-propositions respectively on fundamental epistemic principles. Thus, none of these theories might be simply false, and they might also succeed given the specific explanatory desiderata.

In this paper, I will begin by developing a theory of RID, then using this theory to analyze and reframe the existing debate about DD. There are good prima facie reasons to assume a close connection between DD and RID. Intuitions about DD often center on a form of disagreement that is not resolvable in a rational way, making paradigmatic cases of DD paradigmatic instances of RID. The intuitive connection between DD and RID can be illustrated by considering a paradigmatic case of DD that is rationally irresolvable and small variations of the case, which make the disagreement rationally resolvable. By comparing the cases, the reader is expected to feel a pull towards judging that the resolvable cases are not instances of DD. Disagreement about the age of the Earth between a creationist C and a scientist S is often regarded as a paradigmatic instance of DD. Suppose that C believes that the Earth is 6000 years old because of her bible-based religious belief system whereas S believes that the Earth is several billion years old based on scientific evidence. This kind of disagreement, presented as a paradigmatic case of DD, is usually assumed to be irresolvable, because C and S trust different sources that deliver different verdicts about the age of the Earth.^8^ However, take the following slightly modified cases, which are resolvable variants of this kind of disagreement.

1. Creationist accepts a geological method Suppose that C is a restricted creationist concerning the age of the Earth in the sense that there is one geological method which C accepts that can potentially override the biblical sources. Applying this method delivers that the Earth is several billion years old and the disagreement between C and S is resolved.
2. Scientist accepts a theological source Suppose that there is one passage in the bible such that S accepts that it can override scientific evidence. C and S consult this passage that delivers that the Earth is 6000 years old. The disagreement is resolved.
3. Creationist and scientist accept a ‘neutral’ source Both parties agree to trust a third person concerning information about the age of the Earth, someone who is a religious leader and a trained geologist. Both parties believe what the third person reports and the disagreement is resolved.

Here, we have three modifications of the initial case of DD which differ concerning their resolvability. Do we still think that the parties initially deeply disagreed about the age of the Earth in these three modified cases? Intuitively, I think, they did not deeply disagree in any of these three cases because the disagreement could easily be resolved. Accordingly, we tend to judge that disagreement is not deep if we figure out that it is resolvable or that it has been resolved.

There is a strong tendency to judge that DD cannot be resolved in a rational way, which has to be further specified.^9^ Accordingly, Fogelin (1985) finishes his paper by summarizing that “there are disagreements, sometimes on important issues, which by their nature, are not subject to rational resolution.” This diagnosis is the starting point of the alternative project pursued in this paper, which chooses a different methodological path and aims at reframing the discussion about DD. Many theorists develop a theory about DD which they then use to explain its rational irresolvability. This paper reverses the order of explanation by first developing a theory of RID and then using that theory to explain (away) DD. In the following sections, I will provide a precise definition of RID, which is itself an interesting phenomenon since we tend to think that rational attitudes and procedures can paradigmatically resolve disagreement.^10^ In the final section, I will then suggest replacing the discussion about DD by instead talking about (systematic) RID or that a theory of RID is at least a constitutive part of a theory of DD. The proposed RID-view of DD has a clear methodological advantage over its rivals, since it does not rely on controversial concepts such as “hinge propositions”, “framework propositions”, “fundamental epistemic principles”, or metaphors such as “forms of life”.

Overview: In Sect. 3, I will provide some clarificatory groundwork. I will clarify the concepts of disagreement and resolving disagreement and I will analyze the notion of *rationally resolved* disagreement. In Sect. 4, I will analyze the notions of resolv*able* and irresol*vable* disagreement. In Sect. 5, I will bring the results of Sects. 3 and 4 together and develop the crucial notion of *rationally resolvable and irresolvable disagreement*. Section 6 presents various instances of RID, while Sect. 7 discusses the methodological advantages of a RID-theory of DD over alternatives views on DD.

## Rationally resolved disagreement

Let me start by clarifying the notions of disagreement and resolving disagreement. Two parties, A and B, which can be persons, groups, or institutions, disagree about *p* iff A and B have different doxastic attitudes towards *p*.^11^ According to a more coarse-grained picture, there are three doxastic attitudes that a subject can hold towards *p*, believing that *p*, rejecting that *p* (which is often assumed to be equivalent to believing that ¬*p*) and suspending judgment about *p*.^12^ According to this picture, A and B disagree about *p* iff (1) A believes that *p* and B rejects that *p* or suspends judgment about *p* or (2) A suspends judgment about *p* and B accepts or rejects that *p*. If one opts for a more fine-grained picture of credences, then A and B disagree about *p* if they have different credences concerning *p*.

*Resolving disagreement* is a process where A and B initially disagree about *p* and, because of argumentative interactions, end up having the same doxastic attitude towards *p*.I understand argumentative interactions in a very broad sense, involving, for example, verbal argumentation, non-verbal demonstration, scientific proof, or consulting sources such as witnesses, encyclopedias, or the internet. Disagreement can disappear in various ways, but not all cases of disappearing disagreement might involve potentially rational persuasion. In this paper, I will develop an account of RID. Thus, I will focus on those processes of resolving disagreement that can be regarded as potentially rational.^13^

On our way to a theory of RID, let me provide a more detailed picture about the concept of rationality underlying rational resolvability and irresolvability. Rationality is a rather heterogenous concept, and the views defended about rationality are astonishingly diverse.^14^ For the purposes of this paper, we only need to distinguish an objective concept of rationality from a subjective one. In the case of objective rationality, the subject believes and acts in accordance of what objective rules of rationality dictate, whereas in the case of subjective rationality, the subjects believe and act according to their own standards of rationality, i.e., they believe what they believe to be rational or reasonable.^15^ In terms of beliefs and evidence, we can characterize objective rationality and subjective rationality as follows:

**Objective rationality**

S is objectively rational concerning p and evidence E iff:

S believes that *p* on the basis of E iff E supports *p* (sufficiently) stronger than ¬*p* and

S rejects that *p* on the basis of E iff E supports ¬*p* (sufficiently) stronger than *p* and

S suspends judgment about* p* on the basis of E iff E equally supports *p* and ¬*p*. (This is also the case if E equally neither supports *p* nor ¬*p*.)^16^

**Subjective rationality**

S is subjectively rational concerning *p* and evidence E iff:

S believes that *p* on the basis of E iff S *believes* that E supports *p* (sufficiently) stronger than ¬*p* and

S rejects that *p* on the basis of E iff S *believes* that E supports ¬*p* (sufficiently) stronger than *p* and

S suspends judgment about *p* on the basis of E iff S *believes* that E equally supports *p* and ¬*p*.

The notion of subjective rationality is not meant to require that the subject possesses a complex concept of rationality. Believing that it is good or right to believe that *p* because of E would also suffice. Moreover, S might not even hold an explicit belief about *p* and E and might form beliefs on the basis of E habitually rather than reflectively.

What does it mean to say that two parties resolve disagreement in a rational way? The following definition captures a natural understanding of rationally resolving disagreement:

**Rationally resolving disagreement (objective)**

A and B rationally resolve disagreement about *p* iff they resolve the disagreement by using a method (or set of methods) M that is proper for inquiring into whether *p* is true, and A and B form the same, objectively rational doxastic attitude on the basis of M.

Note that the concept of rationality involved here is a concept of *objective* rationality. A disagreement is rationally resolved in an objective sense when both parties rationally rely on appropriate methods for resolving their disagreement. Disagreement between A and B is irrationally resolved in an objective sense of rationality (a) if it is resolved but A and B did not use a proper method, (b) if A or B do not believe what the proper method indicates, or (c) if A and/or B believe what the proper method indicates but not because of this indication.

Objectively proper methods can include arguing for *p*, presenting an objection against *p*, defending *p* by refuting an objection, or any combination of these elements. Other appropriate methods for resolving disagreement include performing scientific experiments, or observation and demonstration. Furthermore, we can classify methods for rationally resolving disagreement by the sources consulted: these are for example our senses, other reliable persons, technical measurement devices, reasoning, or a combination of these sources. This list is not meant to be complete, but it should suffice to provide a picture of which methods can lead to an objectively rational resolution to a disagreement.

We have distinguished a concept of objective rationality from a concept of subjective rationality. Rationally resolving disagreement in a subjective sense of rationality can be defined as follows:

**Rationally resolving disagreement (subjective)**

A and B rationally resolve disagreement about *p* iff they resolve disagreement by using a method or set of methods M *of which A and B believe* that it is proper for inquiring into whether *p* is true and A and B form the same doxastic attitude on the basis of M (of which they believe that it is rational given M’s indication.)

According to this subjective concept, A and B can rationally resolve disagreement via any method, as long as they both believe that it is appropriate. For example, they can both rationally resolve disagreement about *p* by using a crystal ball if they both believe that this is a proper method. Disagreement is irrationally resolved in a subjective sense (a) if it is resolved by using a method which A or B believes that it is not appropriate for determining whether *p* is true, (b) A or B form a belief that they believe to be irrational on the basis of M’s indication, or (c) A or B does not believe that *p because of* the method that is believed to be proper.

This subjective formulation of rationally resolving disagreement is presumably rather wide according to our understanding of resolving disagreement in a rational way. A more natural understanding relies on objective rationality. However, the concept of rationally resolving disagreement in a subjective sense of rationality will become central later on when we investigate the modal aspect of RID, which can only be plausibly interpreted in a subjective sense.

## Resolvable and irresolvable disagreement

Now that we have two varieties of rationality on the table, I will analyze the notions of resolvable and irresolvable disagreement. Resolvability and irresolvability are modal notions. One way to define resolvable disagreement is in terms of possible worlds: One could say that disagreement between A and B about *p* is resolvable iff A and B disagree about *p* and there is a nearby possible world where A and B do not disagree about *p*. However, this definition is intuitively flawed. Suppose that A and B disagree about *p* and there is nothing that anybody can do to resolve this disagreement. Suppose further there is a nearby possible world where A originally formed a different doxastic attitude towards *p* such that A and B agree about *p*. In this case, we would still say that the disagreement is irresolvable although there is a nearby possible world where A and B agree. The problem of this possible-worlds approach is that it does not capture the procedural, diachronic aspect of resolving disagreement.

Accordingly, one might propose the following definition of resolvable disagreement in terms of possible worlds: Disagreement between A and B about *p* is resolvable iff A and B disagree about *p* and there is a nearby possible *future* world where A and B agree about *p*. However, this definition is also arguably too wide.^17^ Suppose that A and B disagree about *p* at t_1_ but there is a nearby possible world where A is struck by lightning at t_2_ and from this moment on agrees with B about *p*. In this case, we would still hesitate to call the disagreement resolvable as no one, neither A nor B nor someone else, has *done* something to resolve the disagreement. Resolving disagreement means that A and B disagree at t_1_ about *p* but, because of argumentative interactions, agree at a later time t_2_ about *p*. Hence, for disagreement to be resolvable requires that one party is (or both parties jointly are) capable of doing something to resolve it.

Taking these issues into account, resolvable disagreement can be properly specified as follows:

**Resolvable disagreement**

There is resolvable disagreement between A and B about *p* iff A and B disagree about *p* and there is a method M available to A and B such that consulting M leads to agreement between A and B about *p*.

With this understanding of resolvable disagreement, we can now give an account of *irresolvable* disagreement. A and B irresolvably disagree about *p* iff A and B disagree and this disagreement is not resolvable. This is a very general characterization of irresolvable disagreement, which also contains an element of vagueness concerning the required availability of methods. Accordingly, we can distinguish different forms of resolvability and irresolvability.

Practical irresolvability

A and B disagree about *p* and for purely practical reasons there is no method available which can be consulted to resolve the disagreement. Example: A and B disagree about *p* and there is a third person that they both recognize as an authority on *p*, but A and B ran out of gas and cannot visit the third party to ask whether *p* is true.

Temporal irresolvability

A and B disagree about *p* and there is no method currently available which can be consulted to resolve the disagreement. Example: There is a scientific *experimentum crucis* that cannot be performed yet but that would resolve the dispute about *p*.

Fundamental irresolvability

A and B disagree about *p* and there is no potential method available at all (neither now nor at any other time) that can be consulted to resolve the disagreement.

Fundamental irresolvability is irresolvable disagreement in its strongest form. For the purposes of the paper, it is not necessary to select *the* correct definition of irresolvable disagreement. However, it seems intuitively incorrect to say that disagreement is irresolvable just because A and B ran out of gas. Moreover, fundamental irresolvability seems to be a too restrictive form of irresolvable disagreement. Thus, the version of resolvability relevant here lies plausibly somewhere in between. I will provide further specifications whenever it is necessary.

## Rationally resolvable and irresolvable disagreement

So far, I have clarified the notions of rationally resolving disagreement and of resolvable disagreement. In this section, I will put these pieces together to provide an account of RID. I defined two notions of rational disagreement resolution, one that is based on an objective concept of rationality and one that relies on subjective rationality. Accordingly, we can distinguish an objective and a subjective concept of rationally resolvable and irresolvable disagreement.

**Rationally resolvable disagreement (objective)**

There is rationally resolvable disagreement between A and B about *p* in an objective sense if (1) there is a method (or a set of methods) M available to A and B that is proper for inquiring into whether *p* is true and, (2) if M were used to determine whether *p* is true, then A and B would form the same rational doxastic attitude concerning *p* on the basis of M.

**Rationally resolvable disagreement (subjective)**

There is rationally resolvable disagreement between A and B about *p* in a subjective sense, if (1) there is a method (or a set of methods) M available to A and B *of which A and B both believe* that it is proper for inquiring into whether *p* is true and, (2) if M were used to determine whether *p* is true, then A and B would form the same doxastic attitude concerning *p* of which A and B believe that it is rational on the basis of M.

One can also use the notions of objectively and subjectively rational *subjects* to formulate versions of rationally resolvable disagreement. Disagreement about *p* is rationally resolvable in an objective sense if there is an objectively proper method M available for determining whether *p* is true and A and B are *objectively rational* concerning M and *p*, i.e., if M supports that *p*, then A and B believe that *p* because of M, if M supports ¬*p*, then A and B reject that *p*, and if M equally supports *p* and ¬*p*, then A and B suspend judgment about *p*. Rationally resolvable disagreement in an subjective sense can be defined analogously on the basis of subjective rationality and methods that are believed to be rational.

Let us next come to the crucial notion of rationally *ir*resolvable disagreement, RID, in an objective and a subjective sense. In the following, I will identify four possible interpretations of RID, two objective and two subjective. As I will argue, the only plausible understanding of RID is the first of the subjective interpretations. The two potential interpretations of RID in an objective sense are:

1. There is no objectively proper method available to A and B for determining whether *p* is true. In this case, there might be a method such that A and B resolve disagreement, but it is not objectively proper.
2. There is an objectively proper method M available to A and B for determining whether *p* is true but A and B do not agree on the basis of M. For example, even if A and B knew that M indicates that *p*, they nevertheless would not agree about *p* on the basis of M’s indication. In this case, A or B or both are not objectively rational concerning the proper method.

According to this classification, there are two candidate situations for determining objective RID, the lack of an existing proper method or improper doxastic attitudes towards an objectively proper method. Let’s now turn to RID in a subjective sense of rationality. Again, we can distinguish two interpretations:

(3) There is no method available for A and B such that A and B both believe that it is a proper method for inquiring into whether *p* is true.
(4) There is a method available of which A and B believe that it is proper but A or B nevertheless do not form the belief that they believe to be rational on the basis of M’s indication.

There is a connection between the potential reasons (1)-(4). In case (2), there is a proper method M available, but A and B cannot reach agreement via M. The reason can be (2.1) that A and B are subjectively rational concerning M and *p* but have different beliefs about whether M is proper, or (2.2) that they agree that M is proper but A proceeds to base her belief on M whereas B does not. In this case, B is subjectively irrational concerning M. Notably, (2.1) is an instance of (3) and (2.2) is an instance of (4).

I have identified four different candidates for understanding RID. What is the relevant form? Let us have a closer look at (1), where there is no proper method for determining whether *p* is true. Suppose that A and B are perfectly rational. In particular, A and B are objectively rational and do not hold back any evidence. In this case, it is hard to see why lacking a proper method for determining whether *p* is true should lead to irresolvable disagreement because A and B would both just suspend judgment about *p* and agreement is reached.^18^ Thus, (1) is not a plausible interpretation of RID. Note that there is a difference between resolving disagreement and settling a question. Two parties settle a question about *p* if they reach agreement as to whether *p* is true or false. This is also a form of resolving disagreement. However, disagreement can also be resolved without settling a question, namely if A and B suspend judgment about whether *p* is true in the absence of clear evidence in favor of or against it.^19^

Concerning (2)-(4), we can basically distinguish two cases: First, A and B are subjectively rational but have different beliefs about proper methods for determining whether *p* is true (3 and 2.1). Second, A and B have the same beliefs but at least one does not follow them (4 and 2.2). In this case, A or B (or both) fails to be subjectively rational. Not following one’s beliefs about rationality is an epistemic vice on the subjective level. It can be regarded as a form of stubbornness or, as Fogelin calls it, pig-headedness, which is not an instance of DD. These are also not interesting instances of irresolvable disagreement and in particular not of RID. Thus, (4) is not an appropriate analysis of RID. (1) is also inappropriate. (2), however, is either an instance of (3) or of the inappropriate interpretation (4). Consequently, case (3), i.e., RID involving subjectively rational parties, is the interesting form of RID, which I will investigate hereinafter.

Let me next provide a more detailed picture about the kind of subjective rationality relevant for RID. Subjective rationality can be more or less explicit and reflective. On a basic level, subjects follow certain rules or principles of rationality without being able to explicitly articulate them. Take, for example, young children who know how to discuss or argue but are not able to express the underlying principles that they follow. On a more reflective level, subjectively rational subjects can explain their principles of rationality and defend them against objections. For the purposes of this paper, subjects who are subjectively rational on a more reflective level are more interesting, but the points made also apply to less reflective subjects who simply follow implicit rationality principles.

Let me continue spelling out subjective rationality. In some cases, agreement cannot be achieved because one party holds back evidence that she could present in favor of or against *p* or because the party presents misleading evidence. If someone acts this way against her own beliefs about rationality then the party suffers from an epistemic vice that can be characterized as dishonesty. Accordingly, we can define subjective honesty as follows.

**Subjective honesty**

S is subjectively honest concerning *p* and E iff: S presents E in order to settle the question whether *p* is true iff S believes that E is accurate and believes that E is relevant for *p*, i.e., if S believes that E either supports *p* or ¬*p*.^20^

Moreover, we are interested only in subjects who do not aim to deceive the other in some other sense. Thus, only cases where A and B are *subjectively virtuous* in the following sense are interesting cases of RID about *p*:

- **Subjective virtuosity**
- (1)  A and B are subjectively rational concerning *p* and any evidence E,
- (2)  A and B are subjectively honest, and
- (3)  A and B do not aim at deceiving or misleading each other in any other way.^21^

  Note that there is a tension in the results acquired about interesting cases of rationally resolved and rationally resolvable disagreement. When we talk about rationally *resolved* disagreement, we tend to be interested in cases relying on objective rationality. However, RID is the most interesting when it comes to a subjective sense of rationality.^22^

Let me make a final adjustment. The more interesting cases of RID are those where we keep fixed the doxastic attitudes of A and B about rationality. Take the following case. A and B are members of different cultures and disagree about *p*. A and B cannot rationally resolve the disagreement at the moment, but if A entered B’s culture, then A would eventually come to adopt B’s beliefs and standards of rationality via education and thereby the disagreement would be resolved. In some sense, the disagreement is resolvable, nevertheless this is intuitively a case of RID. Hence, when we talk about RID, we tend to keep fixed the beliefs that A and B hold about rationality, i.e., given the beliefs that A and B hold, the disagreement is irresolvable.^23^

## Instances of rationally irresolvable disagreement

In this section, I will further develop the theory of RID. Above, I have characterized RID in terms of methods, which are not available to subjectively rational persons for resolving disagreement. Particularly interesting instances of RID are cases where A and B disagree about specific epistemic features in a systematic way. In this section, I will reflect on further forms of RID, relying on disagreements about evidence broadly conceived, on reliability of sources, and on rational argumentation. Finally, I will reflect on disagreement about supporting evidence and meta-evidence.

### Evidence

Let me first characterize RID in general terms of evidence. In this paper, I will use a broad notion of evidence that includes consulted sources, argumentation, experiments, and so on.^24^ In terms of evidence, RID can be formulated as follows:

**RID about evidence**

A and B rationally irresolvably disagree about *p* iff A and B disagree about *p*, and there is no evidence E available to A and B such that A and B are subjectively virtuous concerning *p* and E and E leads to agreement between A and B about *p*.

If S is subjectively rational concerning E and *p*, then in the absence of any defeaters S believes that *p* because of E iff S believes that E is accurate and that E supports *p*.^25^ Accordingly, we can distinguish disagreement about the accuracy of evidence and disagreement about whether the evidence supports the target proposition. Accordingly, we can distinguish two forms of RID.

**RID about the accuracy of evidence**

If A and B disagree about *p* and are subjectively rational concerning *p* and there is no evidence available to A and B such that A and B agree about its accuracy, then there is RID between A and B about *p*.

**RID about the support of evidence**

If A and B disagree about *p* and are subjectively rational concerning *p* and there is no evidence available to A and B such that A and B agree about whether it supports *p*, then there is RID between A and B about *p*.

In the first case, there is systematic disagreement between A and B about the accuracy of evidence, in the second case about whether the evidences support *p*. These claims hold regardless of whether one of the parties is also objectively rational concerning E and *p*.

### Sources

So far, we have captured the idea of subjective rationality in very general terms of beliefs about evidence. Let me differentiate further the conception of subjective rationality, starting with beliefs about sources. Sources include perception, technical devises, testimony and so on. Let me first address subjective rational behavior concerning sources. There is something irrational about S if S believes *p* because of a source O and believes that O is unreliable.^26^ For example, it is irrational for S to believe that the tank is half-full on the basis of her gas gauge indicating that the tank is half-full if S believes that her gas gauge is unreliable (This does not exclude that it can be rational for S to believe that *p* for other reasons). This is a violation of rationality on a subjective level. S fails according to her own standards of forming beliefs on the basis of reliable sources and not on the basis of unreliable sources. Accordingly, we can define subjective rationality concerning sources as follows:

**Subjective rationality concerning sources**

S is subjectively rational concerning source O iff: In the absence of defeaters, S believes that *p* on the basis of O iff S believes that O is a reliable source in the domain of *p* and believes that O indicates that *p*.

Accordingly, S fails to be subjectively rational concerning O (1) if S believes that *p* on the basis of O although S does not believe that O is reliable or that O indicates that *p* or (2) if S does not believe that *p* on the basis of O although S believes that O is reliable and believes that O indicates that *p*. Based on this concept of subjective rationality concerning sources, we can identify two forms of RID, based on disagreement about the reliability of sources and on what sources indicate.

**RID about the reliability of sources**

If A and B (1) disagree about *p*, (2) are subjectively rational concerning *p,* and (3) there is no source available to A and B such that A and B are subjectively rational about it and agree about its *reliability*, then there is RID between A and B about *p*.

**RID about the indication of sources**

If A and B (1) disagree about *p*, (2) are subjectively rational concerning *p,* and (3) there is no source available to A and B such that A and B are subjectively rational about it and agree about its *indication*, then there is RID between A and B about *p*.

For the purposes of this paper, disagreement about the reliability of sources is more interesting. For example, the case of disagreement between a creationist and a scientist about the age of the Earth presented in Sect. 1 is an instance of disagreement about the reliability of sources. Thus, paradigmatic cases of DD and RID rely on disagreement about the reliability of sources.^27^ There might also be disagreement about what certain sources indicate, like in the case of complex scientific measurement devices, but systematic disagreement about the indication of sources seems rare.

### Argumentation

Various techniques can be used to resolve disagreement, including demonstration, consulting sources, argumentation, and often combinations of these techniques. In this subsection, I will sketch the possibilities and limits of rationally resolving disagreement via argumentation. I understand argumentation as a verbal exchange between two parties A and B with the goal of settling the question whether *p* is true. These verbal exchanges include arguments for or against a proposition, presentations of defeaters, and replies against objections. For the sake of simplicity, I assume that these discursive maneuvers all share the structure of arguments, consisting of a set of premises and a conclusion, which are presented in support of the conclusion. In the context of argumentation, the attitude of subjective rationality can be characterized as follows:

**Subjectively rational arguer**

S is a subjectively rational arguer iff, concerning any argument R,

1. As a speaker, S presents R only if S believes that the premises and the conclusion of R are true and believes that the premises properly support the conclusion and
2. As a hearer, S believes the conclusion of R because of R iff S believes sufficiently many premises and believes that the premises properly support the conclusion.

Suppose now that A and B are subjectively rational arguers and disagree about *q* and A presents an argument R to B. Since, A is a subjectively rational speaker, A believes the premises of the argument and its conclusion and believes that the premises properly support the conclusion. Given that B is a subjectively rational hearer, if B does not believe that the premises are true or does not believe that they properly support the conclusion, then B will not be persuaded. The disagreement remains unresolved. Suppose that A believes that *q* but that B suspends judgment about *q* or rejects it. Suppose further that A believes premises *p*_1_…*p*_n_ and presents an argument based on these premises in support of *q*. If B does not believe the premises, then given that B is subjectively rational, B will not believe that *q* based on A’s argument. The same holds if B does not believe that the premises of A’s argument properly support the conclusion. In the case of evidence, we distinguished between disagreement about the accuracy of evidence and disagreement about the supporting relation between the evidence and the target proposition. Analogously, we can identify two forms of RID about arguments.

**RID about premises**

If A and B disagree about *p* and there is no argument available to A and B such that A and B are subjectively rational arguers and agree about the truth of its premises, then there is RID between A and B about *p*.

**RID about the cogency of arguments**

If A and B disagree about *p* and there is no argument available to A and B such that A and B are subjectively rational arguers and agree about whether the argument’s premises support *p*, then there is RID between A and B about *p*.

Rational resolvability of disagreement between two arguers requires agreement about sufficiently many premises and agreement about the supporting relations between premises and conclusion.

Let me make one more refinement. B can believe the premises of an argument presented by A for two reasons. Firstly, B can already believe the premises prior to A’s presentation or, secondly, B can believe the premises *because of* A’s presentation.^28^ In the second case, B *trusts* A in that B believes the content of A’s utterance because of A’s utterance. This trust might be a simple habit, as with small children who trust their parents, or reflective, if A explicitly and defensibly believes that B is a trustworthy person. Taking these two reasons for believing premises into account, we can say:

If A and B are subjectively rational arguers and disagree about whether *p* is true and there is no argument for or against *p* available such that A and B both believe the premises prior to engaging in argumentation and A and B do not trust each other concerning the premises, then there is RID between A and B.

### Supporting evidence and meta-evidence

Resolving disagreement is a process that can proceed in various steps and on various levels. Disagreement between A and B about whether E is accurate or whether E properly supports *p* need not be the final step of a process aiming at resolving disagreement. A and B can collect *supporting evidence* about whether E is accurate or *meta-evidence* about whether E properly supports *p*, and supporting evidence and meta-evidence for the supporting evidence and the meta-evidence, and so on. However, the requirements for this supporting evidence and meta-evidence for resolving disagreement are always the same. Hence, at some point in a chain of proof, consisting of supporting evidences and/or meta-evidences, there has to be initial agreement about the accuracy of evidence and about the supporting relation. Hence, we can say:

**RID about accuracy of evidence and of supporting evidence**

If A and B are subjectively rational, disagree about *p,* and there is no evidence for *p* and no supporting evidence available to A and B about whose accuracy A and B agree, then there is RID between A and B about *p*.

**RID about supporting relation**

If A and B are subjectively rational, disagree about *p,* and there is no evidence E and no meta-evidence at any level available to A and B such that A and B agree about its support, then there is RID between A and B about *p*.

The first thesis states that A and B cannot rationally resolve their disagreement if they disagree about the accuracy of any evidence, including any supporting evidence. The second thesis makes an analogous claim about disagreement about meta-evidence concerning supporting relations. Moreover, combinations of these factors can also lead to RID.^29^

To sum up: The potential evidence that can be used to resolve disagreement is manifold, including demonstrations, experiments, testimony, and argumentation. Often, disagreement is resolved by using a combination of these different methods. Accordingly, RID can appear due to combinations of factors, including missing agreement concerning the reliability of sources, the cogency of arguments, or the truth of premises.^30^ Accordingly, RID is a complex phenomenon and various forms of RID are possible.^31^

## The RID-view of deep disagreement

RID is an interesting phenomenon since it conflicts with the view that procedures that are rational or believed to be rational are paradigmatic tools for resolving disagreement. However, this paper also aims at investigating whether it is explanatorily fruitful to understand DD as an instance of RID. Call an affirmative answer to this question the *RID-view of DD*. In this final section, I will investigate the prospect of this view. DD is an intuitively introduced phenomenon motivated by intuitions about cases, and there are different views about DD that are defended. However, we have seen that one central tendency is to judge that DD is irresolvable via rational procedures.^32^ The RID-view of DD captures this intuition.

The two central questions concerning DD are Q1, the metaphysical question about its nature, and Q2, the epistemic question about its resolvability. RID is a clearly defined notion, in contrast to the intuitively introduced notion of DD. As a result, the central metaphysical and epistemic questions are both clearly answered for RID. The metaphysical question is addressed by the account of RID provided in this paper and the different instances of RID. The question about the resolvability of RID is by definition answered in the negative. Moreover, it is clearly specified in which sense RID is rationally irresolvable.

In order to get an RID-view of DD off the ground, we need to show that DD falls in the domain of the provided theory of RID. DD is introduced via paradigmatic cases, and there are different accounts of its nature. Consequently, I do not aim at providing a theory of RID that applies to all instances of DD or exactly matches the existing picture of DD, since there is no such unified picture. Nevertheless, in order to defend an RID-theory of DD, paradigmatic cases of DD should also be paradigmatic cases of RID. This is indeed the case. Paradigmatic cases of DD, such as the case of the creationist and the scientist, which are cases of disagreement about the reliability of sources, are also paradigmatic cases of RID.

For evaluating whether the developed RID-view can be fruitfully used in explaining DD, we should gain a clearer understanding of what a theory of DD should achieve. Ranalli (2021, 985) diagnoses four desiderata for a theory of DD:

*Disagreement* It needs to be consistent with the conflict being a *genuine* disagreement.

*Reason-taking* It needs to be consistent with the view that in cases of deep disagreement, the disagreeing parties at least *take themselves* to be *giving reasons* for their views.

*Systematicity* It needs to explain why deep disagreements involve *systematic* disagreement.

*Persistence* It needs to explain why deep disagreements tend to be *persistent* and thus irresolved.

These are intuitively plausible criteria for a theory of DD, and I will use them hereinafter for evaluating different views about the relationship between RID and DD. As I see it, we can distinguish three main views about the relationship between RID and DD:^33^

**The replacement view**

DD simply *is* RID. The provided theory of RID fully explains DD. Consequently, the notion of DD can be eliminated and theorizing about DD can be replaced by theorizing about RID.

**The combination view**

Rational irresolvability is a crucial necessary feature of DD, but it is not its only feature. DD is also deep in some significant sense beyond rational irresolvability. The provided theory of RID explains one crucial aspect of DD, but a full explanation of DD must also contain a theory explaining the systematicity and depth of DD.

**The separation view**

RID and DD are two distinct phenomena, and rational irresolvability is not a necessary feature of DD. The provided theory of RID does not contribute to explaining DD, and a full explanation of DD does not contain a theory of RID.

According to the replacement view, the concept of RID is prior to the concept of DD and the latter can be reduced to the former. The combination view and the separation view, in contrast, assume a conceptual independency (or even a priority) of DD from RID.^34^ Let me evaluate in the following the three alternatives in more detail. I will express strong sympathies for the replacement view for methodological reasons, which do not strictly rule out the other views. I think that the combination view is methodologically inferior to the replacement view, but nevertheless a viable option, but as I will argue, there are no good reasons to opt for the separation view.

### The replacement view

The replacement view has it that DD can be reduced to RID and that analyses of and claims about DD can be replaced by analyses of and claims about RID. There is a crucial difference between this replacement view of DD and alternative theories about DD, such as hinge theories and first principles theories. DD is often understood to be disagreement about a certain type of *propositions*, hinge propositions or propositions about fundamental epistemic principles. RID, in contrast, is determined only by whether the beliefs of two subjectively rational parties sufficiently match, allowing RID to occur concerning any proposition. I regard this result as a methodological advantage of the replacement view over alternative theories. Regardless of which proposition type one spots as the potential subject of DD, either propositions about fundamental epistemic principles or hinge propositions, one has to provide a clear definition of this proposition type in order to define DD. However, both types of propositions are less than crystal clear. There is no wide agreement about what hinge propositions are and how they can be incorporated into a theory of DD,^35^ and fundamental epistemic principles are equally unclear. A paradigmatic fundamental epistemic principle concerns the reliability of one’s own sense apparatus, but which principles concerning the reliability of other sources, such as measurement devices or about complex forms of inductive or abductive inferences, are epistemically fundamental? Conflicting views on these issues can be defended.^36^ The provided account of RID and, consequently, the replacement view, in contrast, do not face these problems, since they do not rely on a particular type of propositions.

Moreover, RID also fulfills other desiderata for DD. (1) RID is clearly a case of genuine disagreement. (2) It is a paradigmatic case of reason-taking, since the two parties act according to their own standards of rationality. (3) Many cases of RID are systematic. In particular, many are concerned with the supporting quality of evidence, the reliability of sources, or the cogency of arguments. (4) RID is obviously persistent, since it is, by definition, rationally irresolvable.^37^

Thus, RID not only has methodological advantages over alternative theories about DD, it also meets the majority of Ranalli’s criteria for a theory of DD. However, there is one restriction. Not all instances of RID are systematic. Let me explain: We can distinguish two forms of RID. The first form centers on disagreements about the *supporting relation* between the evidence and the target proposition, the premises and the conclusion of arguments, or on the reliability of sources; and, second, RID can involve disagreement about the *accuracy* of evidence, of the indications of sources, or the truth of the premises. Disagreement of the first kind is systematic, and paradigmatic instances of DD fall under this category. For example, DD between a creationist and a scientist about the age of the Earth is disagreement about the reliability of sources. Moreover, Lynch (2010, 267) claims that DD is ultimately disagreement over fundamental epistemic principles, which concern reliability of sources, and principles such as induction or abduction, which are closely linked with disagreement about the cogency of arguments. All these cases of systematic DD are clearly also instances of RID. However, we have also spotted instances of RID based on disagreement about the accuracy of evidence or the truth of premises. The systematicity of these types of RID is not guaranteed. A and B might disagree about the accuracy of any potential evidence or the truth of any potential premises, but this disagreement might be ad hoc. Hence, RID can be systematic but there can also be RID that is not systematic. In this sense, the concept of RID is broader than that of DD.

The replacement view suggests replacing theorizing about DD with theorizing about RID. Based on the connection between DD and RID, one could argue for a *simple replacement view* along the following lines. We have a rigorous theory of RID, which is clearer than any existing account of hinge propositions or fundamental epistemic principles. Thus, the replacement view has a clear starting point in analyzing DD that alternative theories lack. This is a notable advantage of the replacement view over rival accounts. Furthermore, RID meets the key features of DD, namely disagreement, reason-taking, and persistence. Moreover, there is no valuable depth in DD to be discovered beyond rational irresolvability. Therefore, theorizing about DD should be replaced by theorizing about RID.

One might object that the simple replacement view ignores that DD is deep and systematic in some relevant sense that RID is not, thereby missing a (or *the*) crucial feature of DD. In response, adherents of the replacement view can defend a more nuanced version, according to which the provided theory of RID allows distinguishing between merely contingent and more systematic forms of RID. For example, RID can be based on merely contingent disagreements about the truth of all premises available, but it can also be based on systematic disagreement (for some fundamental reasons) about proper methods or reliable sources as in the case of the scientist and the creationist. A more nuanced form of the replacement view can then hold that RID can be more or less systematic and that DD is *systematic* RID. Call this the *systematic replacement view*.^38^

One welcome consequence of this approach is that we can incorporate existing theories about hinge propositions and first epistemic principles into a systematic replacement view without entering a debate about *the* correct theory of DD. There can be systematic RID relying on disagreement about epistemic principles and systematic RID relying on disagreement about hinge propositions. These are just further forms of RID, and there is no dispute about *the* metaphysical nature of DD.

The provided arguments for the replacement view are, admittedly, not conclusive. For example, one might object that the order of analysis proposed by the replacement view is mistaken. What we have to analyze first is the metaphysical nature and the depth of DD which are then supposed to explain the rational irresolvability of DD and not vice versa. According to this approach, the replacement view of DD puts the cart before the horse and does not explain the rational irresolvability in terms of DD.^39^ By taking up this objection, one can argue for a combination view or for a separation view about DD and RID.

### The combination view

The combination view holds that rational irresolvability is a crucial necessary condition on DD, but it is not sufficient for DD to obtain. There is a frequently shared intuition that DD is deep and systematic. The combination view addresses this intuition. According to the combination view, rational irresolvability plus depth and systematicity as spelled out by epistemic principles theories or hinge theories about DD are crucial constituents of DD. We have seen that also the systematic replacement view can acknowledge systematicity and rational irresolvability, but various forms of systematicity are just potential features of RID. The systematic replacement view does not face the challenge of providing an independent metaphysical theory about the depth of DD. For the combination view, in contrast, such a theory is inevitable. The combination view can come in a weak and in a strong version. According to the weak version, rational irresolvability and depth (or systematicity) are two distinct features of disagreement that can appear without each other. They are, in this sense independent of each other, and cannot be explained or understood in virtue of each other. DD is then just disagreement that jointly fulfills the two independent criteria. In contrast, the strong version of the combination view holds that DD is rationally irresolvable *in virtue of* its depth or systematicity. In this case, the depth or systematicity of disagreement explains why it is rationally irresolvable by appealing to fundamental epistemic principles or hinge propositions.

Both the systematic replacement view and the combination view assume that DD is rationally irresolvable *and* systematic in some significant sense. However, the priority and order of explanation differs. For the strong combination view, depth and systematicity of disagreement are explanatorily prior to rational irresolvability, i.e., the latter can be explained in terms of the former. The weak combination view treats systematicity and rational irresolvability as independent of each other. The replacement view first develops a theory of RID. For the *systematic* replacement view, systematicity is just a further feature of rational irresolvability, as the simple replacement view ignores systematicity. Both replacement views do not provide a self-contained metaphysical theory about the depth of disagreement.^40^

How can we evaluate the combination view in comparison to the replacement view? There is wide agreement that DD is, in some sense, systematic and rationally irresolvable. This intuition is captured by both combination views, which meet all four of Ranalli’s criteria, including systematicity. However, adherents of DD might want to establish an explanatory connection between rational irresolvability and systematicity. This goal is only achieved by the strong combination view, which *explains* the rational irresolvability of a particular disagreement in terms of systematicity or depth. Given this explanatory desideratum, the weak combination view that simply combines two independent features does not seem very attractive. Accordingly, I will focus on the strong combination view as the main rival of the systematic replacement view.

In comparison, I see three advantages for the systematic replacement view over the strong combination view. First, the theory of RID developed in this paper seems to me clearer than existing theories about hinge propositions or about first epistemic principles, which tend to involve rather vague and unclear notions.^41^ Second, even if one provides a clear theory of hinge disagreement or of disagreement about first epistemic principles, one then has to argue that this is the correct analysis of the nature of DD. I do not see the replacement view in need of an analogous second argumentative step, because disagreement about hinge propositions and about first epistemic principles (and other forms of systematic disagreement) can both be interesting forms of systematic RID. In this respect, the systematic replacement view is more flexible and allows classifying various forms of systematic RID without delivering a single theory about the metaphysical nature of DD. Third, the strong combination view has to explain rational irresolvability in terms of the depth and the systematicity of disagreement. The systematic RID-view, in contrast, need not conversely explain depth in terms of RID. Various forms of depth and systematicity can be additional features of RID.

Admittedly, these methodological considerations do not provide conclusive reasons for favoring a systematic replacement view over a combination view. For instance, one might have an intuition about the explanatory priority of depth and systematicity over rational irresolvability so strong that it overrides all methodological considerations to the contrary. In this case, the provided theory of RID contributes to a combination view at least by clarifying in which sense disagreement can be rationally irresolvable and which kind of rational irresolvability has to be explained by theories of DD. Thus, a theory of RID still fulfills a significant desideratum.

### The separation view

Finally, let me reflect on the third view on the relationship between RID and DD, the separation view. The replacement view and the combination view accept that rational irresolvability is a key feature of DD. Both accounts thereby exclude the possibility that DD is rationally resolvable. However, some authors, such as Feldman (2005) and Matheson (2021), argue that DD is not irresolvable. So let us next come to a view on the relationship between RID and DD that allows for rationally resolvable DD.

The separation view holds that rational irresolvability and systematicity of disagreement are two distinct features that do not entail each other. Thus, there can be RID that is not an instance of DD and vice versa. We can distinguish a weaker separation view holding that *some* instances of DD are rationally resolvable and a stronger view stating that *all* are. Any of these two views entails that all versions of the replacement view and the combination view are false, since they hold that rational irresolvability is a necessary condition on DD.

Some might regard this as an advantage of the separation view, given the arguments presented in favor of the rational resolvability of DD. One advocate of the view that DD is rationally resolvable is Feldman. Feldman (2005, 16) presents the following definition of a rational resolution of disagreement:

RR2 There is a rational resolution of a disagreement available when there is some way of presenting arguments and evidence to which the rational response is a resolution of the disagreement (i.e., there is some way of presenting arguments and evidence that should lead to a resolution).

Based on this definition, Feldman argues that DD is resolvable in the sense that there is a rational reaction for both parties available such that the disagreement is resolved. Feldman admits that issues that are subject to DD are often fundamental and, therefore, hard to decide. However, he stresses the point that also suspension of judgment can be a rational resolution of disagreement and that, in many cases, a rational reaction for persons confronted with DD is to suspend judgment about the target proposition. If both parties suspend judgement, then they hold the same doxastic attitude concerning the target proposition and the disagreement is resolved. What Feldman seems to have in mind when talking about DD are cases of deep philosophical disputes, which are hard to resolve. However, also other rational resolutions to DD are possible, where one of the parties should rationally adopt the view of the other.^42^

Feldman is right that DD is resolvable in this particular sense, but note that RR2 expresses a different sense of rational resolvability than the one developed here. According to Feldman, DD is resolvable in the sense that we can give a conceptual analysis of necessary and sufficient conditions of rationally resolving disagreement in an *objective* sense of rationality. Matheson (2021, 9) argues in a similar spirit that DD is resolvable because “what one should believe in a deep disagreement is what the true fundamental epistemic principles dictate that they should believe.”^43^ I argue, in contrast, that for *subjective* reasons, these conditions cannot be fulfilled by two subjectively rational parties who deeply disagree, e.g., about the truth of all available premises or about the reliability of sources. In this sense, RID is practically irresolvable. Take the case of DD between a creationist and a scientist about the age of the Earth. There is an objective (or normative) rational resolution to this disagreement in Feldman’s sense, namely accepting what the scientific evidence recommends. However, the DD is rationally irresolvable in a subjective sense since both parties, given that they are subjectively rational, will not reach agreement. There can be an objective rational resolution to DD available without there being a subjective rational resolution. Thus, Feldman’s and Matheson’s positive views about the rational resolvability of DD in an objective sense do not contradict the view that DD is rationally irresolvable in the subjective sense defended here.^44^ Hence, they are not proponents of the separation view as specified here.

I do not know of any other plausible view about the rational resolvability of DD. For this reason, I currently do not see any motivation for accepting the separation view that rational irresolvability in a subjective sense and depth are two distinct features that should be strictly separated. Accordingly, I regard the separation view as the least plausible theory about the relationship between RID and DD. However, even if one opts for a separation view, then the provided theory about RID can contribute to clarifying this view.^45^

## Conclusion

This paper has offered a theory of RID. We can distinguish between objective and subjective forms of rationality. However, RID can only be formulated in terms of subjective rationality, i.e., two parties disagree in a way that is rationally irresolvable if they hold different views about rationality. Various forms of RID have been distinguished, based on different views about evidence, about the reliability of sources, and about cogent argumentation. One central motivation for developing a theory of RID is its potential application to DD, as existing theories of DD face certain shortcomings. In contrast, an RID-view of DD provides a clear definition of the relevant kind of disagreement and avoids these shortcomings. Moreover, this theory meets plausible explanatory desiderata for a theory of DD and also covers paradigmatic cases of DD. For these reasons, it is reasonable to replace the ongoing discussion about DD with (or at least accompany it with) a theory of RID.

## Footnotes

1 Page numbers refer to reprinted version in *InformaI Logic* 25(1), (2005), 3–11.

2 See Ranalli (2021).

3 A Wittgensteinian approach is also defended by Fogelin. However, Fogelin (1985, 9) thinks that DD does not rely on isolated propositions but instead on a “whole system of mutually supporting propositions (and paradigms, models, styles of acting and thinking)”. This account faces the same problem of specifying the kind of mutually supporting relations relevant for DD. For an extensive analysis of hinge epistemology in the context of DD, see Ranalli (2020) and Kusch (2021). For a further development of hinge epistemology, see Coliva (2015), and for a critical discussion of hinge epistemological accounts of DD, see Siegel (2021).

4 Aikin (2021) analyzes, in a similar spirit, DD as being based on regress problems and the problem of the criterion.

5 This holds, for example, for the case of disagreement between a creationist and a scientist about the age of the Earth discussed below. For the distinction between fundamental and derived epistemic principles, see Matheson (2021).

6 For an alternative, positive view on the resolvability of DD, see Matheson (forthcoming). I will reflect on the problems of these positive views in Sect. 7.

7 For a more detailed methodological criticism of existing accounts of DD, see Melchior (forthcoming a).

8 See Lynch (2010) and Ranalli (2021).

9 Usually, theories on DD also assume that DD is rationally irresolvable because it is systematic in some specific way. In Sect. 7, I discuss two potential relationships between systematicity and rational irresolvability–that systematicity is a feature of some instances of rational irresolvability and that DD is rationally irresolvable in virtue of its systematicity. I will also critically reflect there on views holding that DD is rationally resolvable.

10 Ranalli (2021) is skeptical about the project of developing a theory of RID when he claims: “One immediate thing to note is that the rational irresolvability of the disagreement is not a plausible constraint on a satisfactory theory of deep disagreement. […] Notice that ‘rational irresolvability’ is multiply ambiguous: we might have in mind that (1) there’s no epistemically rational way to resolve the disagreement; or that (2) there’s no practically rational way to resolve the disagreement; or further still that (3) the disagreement isn’t resolvable by way of argumentation and the exchange of reasons.” As we will see, the provided theory can address all of these issues.

11 Moreover, disagreement between three or more parties is possible, but for the sake of simplicity, I will focus in this paper on the case of two parties. However, the results can *mutatis mutandis* be generalized to more than two parties.

12 For a recent discussion on judgment suspension, see McGrath (forthcoming).

13 Thus, resolving disagreement excludes cases of disappearing disagreement where A and B initially disagree about *p* and A changes her doxastic attitude without any interaction with B. Moreover, A might reach agreement with B via coercion or intoxication, but these are also not the cases we are interested in here. For the purposes of this paper, it is not necessary to provide a more detailed account of argumentative interactions.

14 See Rysiew (2012) who notes that since rationality “is a term that is used in so many ways, and with regard to such a wide range of topics and subjects, it does not admit of any neat analysis.” Fogal and Worsnip (2021) distinguish theories of rationality along two dimensions; first, concerning whether reasons are subjective or objective. Second, they distinguish theories about structural rationality, which understand rationality as some kind of coherence, from theories about substantial rationality, which usually interpret rationality as some kind of responsiveness to reasons or to evidence. For recent detailed theories on rationality, see Wedgwood (2017) and Comesaña (2020). For a discussion of different conceptions of rationality, see Worsnip (2018) and Fogal and Worsnip (2021).

15 One might object that subjective rationality is not really a form of rationality, since it is only believed to be one. In this paper, I do not commit myself to any specific view about the nature of rationality. I just use objective and subjective rationality as labels for distinguishing actual rationality from what one believes to be rational. This distinction is central for the purposes of this paper, regardless of whether subjective rationality is a form of rationality or only an alleged one. However, see Schroeder (2004), who provides an account of objective and subjective instrumental rationality, which comes very close to the conception of objective and subjective rationality defended here. It should also be noted that the version of subjective rationality defended here differs from typical notions of subjective *reasons*, since it refers to beliefs *about* evidence and not to beliefs *as* evidence. Moreover, the notions of objective and subjective rationality defended here are determined by the evidence-responsiveness of the subject. However, rationality is also often understood in terms of coherence. I do not aim to provide a full account of rationality here. Rather, I am only interested in the aspect of rationality that is crucial for RID.

16 For the purposes of this paper, I can leave open when the evidential support is sufficiently stronger. Furthermore, the principles of objective and subjective rationality can also be reformulated in more fine-grained terms that apply to credences.

17 A further problem of defining resolvable disagreement merely in terms of possible worlds is that it can be the case that there is a method available for resolving disagreement but there is no nearby possible world where A and B use this method. In this case, we would still tend to judge that the disagreement is resolvable in some relevant sense, although there is no nearby possible world where it is resolved.

18 Except perhaps for some tricky cases of peer disagreement or epistemic paradox, where a body of evidence recommends both *p* and ¬*p*.

19 See on this issue Feldman (2005).

20 Here issues of practical limitation could be considered, for example subjects cannot present arbitrarily many pieces of evidence, even if they think that is can support *p* or ¬*p*. However, these issues are not relevant for the purposes of this paper.

21 The listed attitudes are only meant as a first approach. Perhaps further necessary conditions will have to be added to specify the relevant cases.

22 For an alternative taxonomy distinguishing rational resolvability, rational response, and rational persuasion, see Ranalli (2020). Ranalli’s concept of rational resolvability is a version of what we have called objective rational resolvability. For an alternative taxonomy, see also Matheson’s (forthcoming) concepts of strong rational resolutions and weak rational resolutions.

23 Notably, the presented theory of RID does not presuppose any particular theory about epistemic justification or knowledge.

24 Thus, I do not use the notion of evidence in any specific sense. In particular, I do not claim that evidence is factive. See Williamson (2000).

25 Again, these beliefs about evidence can be implicit or explicit.

26 For this reason, bootstrapping is not a persuasive or rational method of settling the question whether a source is reliable. For a discussion on this aspect of bootstrapping, see Davies (2004), Pryor (2004 and 2012), Markie (2005), and Melchior (forthcoming b).

27 For further discussions of bootstrapping and DD, see Lynch (2010) and Melchior (forthcoming b).

28 There might also exist other cases, for example that B is hit by an object during A’s argumentation and believes the premises of A’s argument because of this impact. However, these are not relevant cases for the purposes of this paper.

29 The most general case about this type of RID can be made in terms of supporting evidence and meta-evidence, but we also had a closer look at the more specific cases of sources and of arguments. The above theses can be mutatis mutandis reformulated for these more specific cases.

30 I argue in Melchior (forthcoming b) that similar structural problems also hold for disagreement with a skeptic and that, therefore, classical skeptical problems involving regresses, meta-regresses, and bootstrapping can be reinterpreted as problems of resolving DD with a skeptic.

31 I do not claim here that I provided a complete list of different instances of RID. For example, RID might also arise due to conceptual incommensurability.

32 Exceptions that I will discuss in this section include Lugg (1986), Feldman (2005), and Matheson (2021).

33 I assume that various intermediate positions are possible.

34 As I will argue, any theory about the relation between RID and DD is compatible with the view that there is DD. Thus, one need not be a pessimist about DD to theorize about RID. Only adherents of a simple separation view will find theorizing about DD superfluous.

35 See Ranalli (2020).

36 Moreover, Lagewaard (2021) argues that Lynch’s notion of DD is too narrow since paradigmatic cases of DD rely on derived and not on fundamental epistemic principles. She suggests that ‘fundamental epistemic principle’ and DD are gradable notions. RID, as defended here, is not gradable. Accordingly, the RID-view of DD avoids notions such as ‘deeper disagreement’ and ‘relatively fundamental epistemic principle’ as used by Lagewaard.

37 One can object that RID itself fulfills the criteria of DD, disagreement, reason-taking, persistence, and often also systematicity, but the replacement view does not meet Ranalli’s criteria for a *theory* about DD since it does not *explain* systematicity and persistence as Ranalli demands. I will take up this objection below.

38 One might object that the replacement view does not meet Ranalli’s criteria since it does not properly explain why the target disagreement is systematic and why it is persistent and thus irresolved. I agree with this diagnosis but do not find this objection very persuasive. I think that an account of DD should be about a form of disagreement that fulfills these four criteria, but I do not see any need of further explaining systematicity and persistence. However, if one does not share this intuition, then one will probably find the combination view more plausible.

39 I am thankful to a reviewer for pressing me on this point. For a similar line of argumentation, see also Ranalli (2021), who suggests that we should develop a theory about the nature of DD that is prima facie open concerning the question whether DD is rationally resolvable.

40 In some borderline-cases, it might not be easy to classify a specific theory, in particular whether it is a systematic replacement view or a weak combination view, but the basic idea behind the classification should be sufficiently clear.

41 For an overview of problems for both accounts, see Ranalli (2021).

42 For example, two parties A and B can deeply disagree about* p* even if A is a perfect reasoner and B is systematically mistaken about fundamental principles of reasoning. Given that A knows about B’s epistemic vices, it seems false to claim that A should rationally suspend judgment about *p*. In this respect, there is a crucial difference between DD and peer disagreement, since A need not acknowledge B as an epistemic peer.

43 The resolvability of DD is also defended by Lugg (1986), but he seems to have in mind a different conception of DD than the one currently dominant in the debate.

44 Notably, Feldman also discusses a subjective concept of rational resolvability, which he criticizes and rejects. However, we have seen that this version of rational resolvability is the crucial one in the context of DD.

45 One might wonder whether RID is the only alternative for replacing DD. For example, one might suggest replacing DD by evidentially imperfect disagreement, where the two disputants evaluate the evidence in different ways or even disagree about what counts as evidence, or by systematic disagreement, or by a combination of these views. One could argue that such an alternative replacement view has the advantage of incorporating resolvable disagreement. I do not claim that the RID-view is the only potential view for replacing DD on the market, and the fruitfulness of alternative replacement accounts depends on how they are spelled out in detail. However, I find rational irresolvability *the* significant feature of the discussion about DD, and cases of irresolvable disagreement due to different views about evidence more significant than resolvable cases of disagreement about evidence. Moreover, I do not find the existing accounts about the resolvability of DD to be convincing. For these reasons, I regard the RID replacement view as the most promising one. However, if one opts for an alternative replacement view, then the proposed theory of RID can still contribute to it by explaining in which particular sense DD is irresolvable or resolvable. I am thankful to an anonymous referee for this suggestion.
